# Selective Overexpression of Collybistin in Mouse Hippocampal Pyramidal Cells Enhances GABAergic Neurotransmission and Protects against PTZ-Induced Seizures

**DOI:** 10.1523/ENEURO.0561-20.2021

**Published:** 2021-07-12

**Authors:** Shanu George, Shaun James, Angel L. De Blas

**Affiliations:** Department of Physiology and Neurobiology, University of Connecticut, Storrs, CT 06269

**Keywords:** AAV, collybistin, GABA, gephyrin, PTZ

## Abstract

Collybistin (CB) is a rho guanine exchange factor found at GABAergic and glycinergic postsynapses that interacts with the inhibitory scaffold protein, gephyrin, and induces accumulation of gephyrin and GABA type-A receptors (GABA_A_Rs) to the postsynapse. We have previously reported that the isoform without the src homology 3 (SH3) domain, CBSH3–, is particularly active in enhancing the GABAergic postsynapse in both cultured hippocampal neurons as well as in cortical pyramidal neurons after chronic *in vivo* expression in *in utero* electroporated (IUE) rats. Deficiency of CB in knock-out (KO) mice results in absence of gephyrin and gephyrin-dependent GABA_A_Rs at postsynaptic sites in several brain regions, including hippocampus. In the present study, we have generated an adeno-associated virus (AAV) that expresses CBSH3– in a cre-dependent manner. Using male and female VGLUT1-IRES-cre or VGAT-IRES-cre mice, we explore the effect of overexpression of CBSH3– in hippocampal pyramidal cells or hippocampal interneurons. The results show that: (1) the accumulation of gephyrin and GABA_A_Rs at inhibitory postsynapses in hippocampal pyramidal neurons or interneurons can be enhanced by CBSH3– overexpression; (2) overexpression of CBSH3– in hippocampal pyramidal cells can enhance the strength of inhibitory neurotransmission; and (3) these enhanced inhibitory synapses provide protection against pentylenetetrazole (PTZ)-induced seizures. The results indicate that this AAV vector carrying CBSH3– can be used for *in vivo* enhancement of GABAergic synaptic transmission in selected target neurons in the brain.

## Significance Statement

Excessive or imbalanced excitation in the hippocampus can result in acute or chronic pathologic conditions, such as seizures, epilepsy, and learning impairments. It is therefore important to uncover target genes that can be manipulated to restore or prevent this imbalance. This study uses a novel adeno-associated virus (AAV) to express collybistin (CB)SH3– in select cells of the hippocampus. We have found that overexpression of this protein enhances GABAergic inhibitory synaptic transmission. The results also bring attention to CB as a possible target for therapeutic intervention aimed to restore the balance between excitation and inhibition.

## Introduction

Phasic inhibitory neurotransmission in the central nervous system is accomplished through the action of the neurotransmitters GABA or glycine acting on ligand-gated channels which primarily cluster at postsynaptic sites. GABA type-A receptors (GABA_A_Rs) concentrate at postsynaptic sites through interactions with postsynaptic proteins, including anchoring on the inhibitory scaffolding protein, gephyrin (Kneussel et al., 1999; Tretter et al., 2008, 2011; Saiepour et al., 2010; Mukherjee et al., 2011). Collybistin (CB) is a guanine exchange factor that interacts with gephyrin, inducing the recruitment of gephyrin and CB, itself, to GABAergic postsynapses (Kins et al., 2000; Grosskreutz et al., 2001; Reddy-Alla et al., 2010). Rodents express several isoforms of CB that differ in C termini (CB1, CB2, CB3), but can also either possess or lack a regulatory src homology 3 (SH3) domain (Kins et al., 2000; Harvey et al., 2004). Isoforms that possess the SH3 domain (CBSH3+) are in an auto-inhibited conformation, thus preventing the pleckstrin homology (PH) domain of CBSH3+ from binding to phosphoinositides of the neuronal membrane (Soykan et al., 2014; Papadopoulos et al., 2015; Ludolphs et al., 2016) unless auto-inhibition is removed through the binding of neuroligin-2 (NL2) or GABA_A_R subunit-α2 to the SH3 domain, or the small GTPase, TC10, to the PH domain (Poulopoulos et al., 2009; Saiepour et al., 2010; Mayer et al., 2013; Papadopoulos et al., 2017; Nathanson et al., 2019). Isoforms that lack the SH3 domain (CBSH3–), are not constrained in this fashion, inducing the formation of submembranous gephyrin clusters, as demonstrated in HEK and COS-7 cells (Kins et al., 2000; Harvey et al., 2004; Poulopoulos et al., 2009).

Studies of CB knock-out (KO) mice reveal that loss of CB results in severe disruption to clustering of gephyrin and GABA_A_Rs in certain brain regions, including the hippocampus (Papadopoulos et al., 2007, 2008). This results in decreases in GABAergic neurotransmission, deficiencies in synaptic plasticity, increased levels of anxiety, and impaired spatial learning in CB KO mice. In humans, a mutation in *ARHGEF9*, the gene that encodes CB, was first identified in a patient with epileptic encephalopathy (Harvey et al., 2004) but additional pathogenic mutations have since been identified in patients, contributing to a spectrum of ailments that include epilepsy, hyperekplexia, intellectual disability, anxiety, autism, and schizophrenia (Marco et al., 2008; Kalscheuer et al., 2009; Lesca et al., 2011; Shimojima et al., 2011; Lionel et al., 2013; Long et al., 2015; Bhat et al., 2016; Klein et al., 2017; Wang et al., 2018; Aarabi et al., 2019; Chiou et al., 2019; Yao et al., 2020).

Overexpression of gephyrin or GABA_A_Rs does not significantly affect synaptic GABA_A_R or gephyrin clustering in cultured hippocampal neurons (Chiou et al., 2011). However, it has been demonstrated that overexpression of CBSH3– in cultured hippocampal neurons induces the formation of large synaptic gephyrin and GABA_A_R clusters (Kalscheuer et al., 2009; Chiou et al., 2011; Tyagarajan et al., 2011; Soykan et al., 2014). In the present study, we examined the effect of overexpressing a constitutively active isoform of CB, CB2SH3–, in selected cells of the dorsal hippocampus in adult mice. By overexpressing CB2SH3– in specific neuron-types, and in adulthood, we aimed to address whether the increase in CB2SH3– expression enhances the postsynaptic accumulation of gephyrin and GABA_A_Rs, GABAergic synaptic transmission in the developed hippocampus, and whether it can effectively protect against pentylenetetrazole (PTZ)-induced acute seizures.

## Materials and Methods

### Animals

All animals included in this study were treated following protocols approved by the Institutional Animal Care and Use Committee (IACUC) at the University of Connecticut and follow National Institute of Health guidelines. Both male and female mice of VGAT-IRES-Cre (JAX: Slc32a1tm2(cre)Lowl/J; stock #016962) or VGLUT1-IRES2-Cre (JAX: B6;129S-Slc17a7tm1.1(cre)Hze/J; stock #023527) background were used.

### Materials

For immunofluorescence studies, mouse mAb gephyrin (1:200; RRID: https://scicrunch.org/resolver/AB_887717), guinea pig anti-VGAT (1:1000; RRID: AB_887873), and rabbit anti-CB (1:1000; RRID: AB_2619977), were from Synaptic Systems. The rabbit anti-CB antibody detects both CBSH3– and CBSH3+ isoforms (Soykan et al., 2014; George et al., 2021). Mouse anti-HA (1:1000; RRID: AB_10063630) was from Covance. The rabbit antibody against GABA_A_R-subunit γ2 (1:25; RRID: AB_2314477; to amino acids 1–15: QKSDDDYEDYASNKT) was raised and affinity-purified (on immobilized antigen peptide) in our laboratory. In cultured hippocampal neurons and in brain tissue, this antibody shows clustered labeling that colocalizes highly with that of antibodies to other GABA_A_R subunits and to gephyrin. It also shows apposition to glutamate decarboxylase (GAD)-containing terminals. It has been described and characterized elsewhere (Khan et al., 1994; Christie and De Blas, 2003; Charych et al., 2004a,b; Li et al., 2005b, 2007, 2010, 2012; Serwanski et al., 2006; Yu et al., 2007, 2008; Chiou et al., 2011; Jin et al., 2014; Fekete et al., 2015, 2017). Species-specific anti-IgG secondary antibodies were raised in goat and labeled with Alexa Fluor 568 or 647 (Invitrogen). PTZ (catalog #18682) used for behavioral studies was from Cayman Chemicals. For electrophysiological studies, tetrodotoxin (TTX; catalog #T-550) was from Alomone Labs; CNQX (catalog #C127) and picrotoxin (catalog #P1675) were from Sigma.

### Construction and production of AAV9/hSyn-DIO-HA-CB2SH3()-IRES-mCitrine

The cDNA for the constitutively active isoform of CB tagged with HA (HA-CB2SH3–) was cloned into the pAAV2-hSyn-dF-HA-KORD-IRES-mCitrine plasmid (gift from Bryan Roth; Addgene plasmid # 65 417; http://n2t.net/addgene:65417; RRID: https://scicrunch.org/resolver/Addgene_65417) to replace HA-KORD. The pAAV2-hSyn-DIO-HA-CB2SH3(-)-IRES-mCitrine was sent to the UNC Vector Core for AAV9 production (Zhu et al., 2014). This plasmid has been submitted to Addgene (https://www.addgene.org/160069/).

### Viral infusion *in vivo*

Adult mice (five to eight weeks old) were anesthetized with isoflurane and placed into a stereotactic frame to prevent any head movement. The viral constructs, AAV/9-hSyn-DIO-HA-CB2SH3(-)-IRES-mCitrine (HACB2-AAV) or AAV/5-hSyn-DIO-EGFP (EGFP-AAV; UNC Vector Core; Roth Lab) were injected into the dorsal hippocampus (from bregma: anterior-posterior: −2.0, medial-lateral: ± 1.6, dorsal-ventral: −1.6) using a 1-μl Hamilton syringe at a rate of 20 nl/min, followed by 5 min before retraction of the syringe. Volumes used were 250 nl unilaterally for immunohistochemical studies and 500 nl bilaterally for electrophysiological and behavioral studies (titers: 7.2 × 10^12^ vg/ml EGFP-AAV; 7.1 × 10^12^ vg/ml HACB2-AAV). After surgery, mice were returned to their cages for two to four weeks of recovery before use for behavioral, electrophysiology, or immunohistochemical study.

### Tissue preparation, immunohistology, and image acquisition

**Figure 1.**
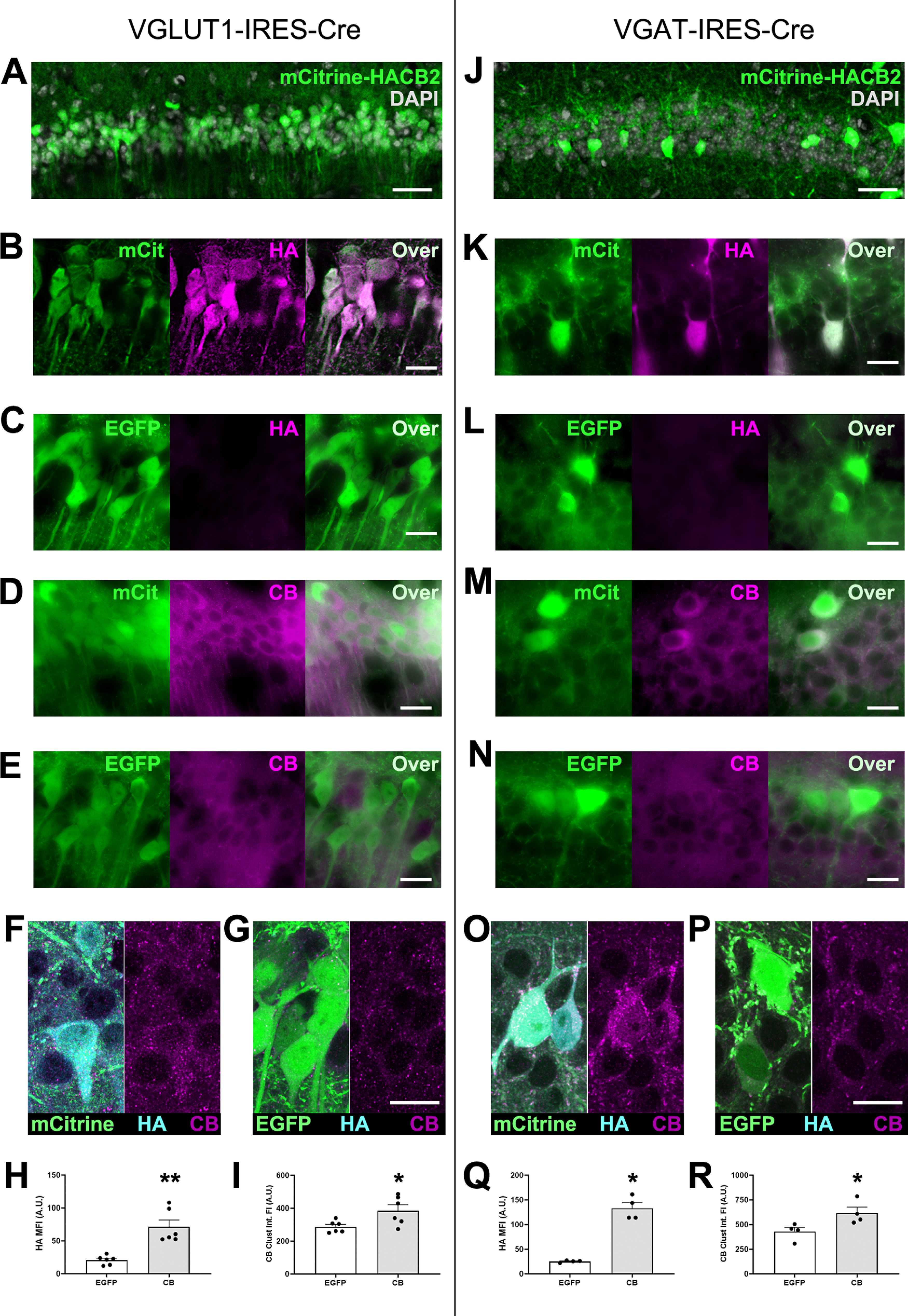
Injection of hippocampal CA1 with AAV-DIO-HA-CB2SH3(-)-IRES-mCitrine results in overexpression of mCitrine and HA-CB2SH3(-) in a cre-dependent manner. ***A–I***, The mCitrine-HACB2-AAV (***A***, ***B***, ***D***, ***F***) or a control EGFP-AAV (***C***, ***E***, ***G***) were injected into CA1 of VGLUT1-IRES-cre mice, resulting in expression of mCitrine or EGFP in the glutamatergic pyramidal cell, and images were taken with epifluorescence microscopy (***A–E***) or confocal microscopy (***F***, ***G***). In mCitrine-HACB2-AAV-injected mice, the transduced pyramidal cells expressing mCitrine also overexpressed HA-CB2SH3–, as detected by Ms anti-HA (***B***, ***F***) and Rb anti-CB (***D***, ***F***). In CA1 of EGFP-AAV-injected mice, HA was not detected (***C*, *G***), and only a low endogenous level of CB (***E***, ***G***) was visible. Quantification from confocal images for mean intensity of HA immunofluorescence in SP is presented in ***H***, and integrated intensity of CB immunofluorescent clusters is presented in ***I***. ***J–R***, The mcitrine-HACB2-AAV (***J***, ***K***, ***M***, ***O***) or a control EGFP-AAV (***L***, ***N***, ***P***) were injected into CA1 of VGAT-IRES-cre mice, resulting in overexpression of mCitrine or EGFP in GABAergic interneurons and images were taken with epifluorescence microscopy (***J–N***) or confocal microscopy (***O–P***). In mCitrine-HACB2-AAV-injected mice, the transduced interneurons expressed mCitrine and overexpressed HA-CB2SH3–, as detected by Ms anti-HA (***K***, ***O***) and Rb anti-CB (***M***, ***O***). In CA1 of EGFP-AAV-injected mice, HA was not detected (***L***, ***P***), and only a low endogenous level of CB (***N*, *P***) was visible. Quantification from confocal images for mean intensity of HA immunofluorescence in interneurons is presented in ***Q***, and integrated intensity of perisomatic CB immunofluorescent clusters is presented in ***R***. Scale bars: 25 μm (***A***, ***J***) and 10 μm (***B–G***, ***K–P***). Asterisks in the quantification graphs indicate significance using Mann-Whitney U test, *p<0.05, **p<0.01.

Three weeks after unilateral viral injection, mice were deeply anesthetized with an overdose of ketamine/xylazine (60/8 mg/kg) and cardiac perfusion was performed with 0.12 m phosphate buffer (PB; pH 7.4) followed by 4% PLP fixative in 0.12 m PB (4% paraformaldehyde, 1.37% lysine, and 0.21% sodium periodate). Brains were cryoprotected with 30% sucrose, frozen, and sectioned with a freezing microtome (25 μm thick) and stored in 0.02% sodium azide in 0.1 m PB, pH 7.4 at 4°C. Immunofluorescence was performed as described elsewhere (Li et al., 2005a; Fekete et al., 2017; Miralles et al., 2020). Briefly, free‐floating brain sections were incubated with 5% normal goat serum, 0.3% Triton X‐100 in 0.1  m PB for 1 h at room temperature. Sections were then incubated for 2 d in primary antibody cocktails raised in different species in 2% NGS/0.3% Triton X‐100 in 0.1 m PB at 4°C. Sections were washed and incubated in a mixture of fluorophore‐conjugated secondary antibodies in 2% NGS/0.3% Triton X‐100 in 0.1 m PB for 1 h at room temperature. When necessary, a DAPI counterstain (200 nm) was applied following a wash after secondary incubation. Sections were then washed again and mounted onto gelatin‐coated glass slides with Prolong Gold Antifade mounting solution (Invitrogen). Confocal images of brain sections were acquired using an A1R laser scanning confocal microscope (Nikon Instruments) with a Plan Apo VC 60×/1.4 oil immersion objective. For the quantifications presented in Figure 1. The images were collected with a LSM 800 confocal laser-scanning microscope (Zeiss) with a 63×/1.4 oil-immersion objective. The pinhole was set at 1.2 Airy units. Images were collected as single optical sections. The low‐magnification images of brain sections for EGFP/mCitrine/DAPI were collected by epifluorescence with a Plan Fluor 10×/0.3 objective.

### Electrophysiological recordings

Mice were anesthetized by administration of ketamine (375 mg/kg, i.p.) and xylazine (25 mg/kg, i.p.) and rapidly decapitated; cortex was removed, and slices (250 μm) were cut using a microslicer (DSK 1500E; Dosaka) in ice-cold substituted Ringer solution containing the following: 83 mm NaCl, 26 mm NaHCO_3_, 1.25 mm NaH_2_PO_4_, 2.5 mm KCl, 22 mm dextrose, 72 mm sucrose, 0.5 mm MgCl_2_, 3.3 mm CaCl_2_, 1.3 mm Na-ascorbate, and 0.6 mm Na-pyruvate. Slices were incubated for 30 min at 37°C and kept at room temperature in a normal Ringer’s solution containing: 140 mm NaCl, 3 mm KCl, 2 mm MgCl_2_, 2 mm CaCl_2_, 10 mm HEPES, and 10 mm glucose. Both substituted and normal Ringer’s solutions were bubbled with 95% O_2_ and 5% CO_2_ (pH 7.3).

Individual slices containing the CA1 hippocampus were transferred to a recording chamber mounted on a fixed-stage microscope (Olympus BX5.1WI) and perfused continuously (∼2 ml/min) with normal Ringer’s solution (equilibrated with 5% CO_2_; pH 7.3). Transfected CA1 hippocampal neurons were visually identified by fluorescent signal from EGFP or mCitrine. All recordings were made with an Axopatch 200B patchclamp amplifier, digitized with a Digidata 1322A A/D converter, and recorded using pCLAMP 10.0 software (Molecular Devices). Recordings were obtained at room temperature (∼22°C) with patch electrodes pulled from borosilicate glass capillaries (Harvard Apparatus) on a two-stage puller (P-97; Sutter Instrument) to a DC resistance of 5–7 MΩ when filled with a pipette solution containing the following: 130 mm CsCl, 8.5 mm NaCl, 5 mm HEPES, 4 mm MgCl_2_, 4 mm Mg2-ATP, 0.3 mm Na-GTP, and 0.6 mm EGTA. Electrode tips were coated with Sylgard 184 (Dow Corning). A gigaohm seal (≥1 GΩ) was achieved using a cell-attached voltage-clamp configuration with holding potential matched to the resting membrane potential (V_hold_ = −70 mV) and with no current generated by the amplifier (I_amp_ = 0 pA). Whole-cell access (≤20 MΩ) was obtained to characterize spontaneous synaptic input. Spontaneous mIPSCs were characterized in the presence of bath applied CNQX (10 μm) and TTX (0.5 μm), and events were confirmed with bath application of picrotoxin (100 μm). mIPSCs were analyzed using Clampfit 10.7 and detected with characteristic kinetics (fast rising phase followed by a slow decay). Each automatically detected event was also visually inspected to exclude false positives. Recordings were discarded if Ra varied 10% during an experiment, and capacitance and Ra compensation (70%) were used to minimize voltage errors.

### Behavioral study

Preliminary trials were performed on naive mice to determine dose of PTZ that would reliably induce acute seizures while minimizing mortality. Three weeks after bilateral viral infusion, mice were subjected to behavioral study. Mice were given a single injection of PTZ (40 mg/kg, i.p.) to induce acute seizures. The mice were then placed into a cage and behavior was observed. Seizures were scored according to a modified Racine scale (Racine, 1972; Lüttjohann et al., 2009; Singh et al., 2016) with the following scores: 1, sudden behavioral arrest; 2, facial jerking; 3, myoclonic jerks; 4, clonic seizure, sitting; 5, clonic, tonic‐clonic seizure, lying on belly; 6, clonic, tonic‐clonic seizure with loss of posture and wild jumping; with notation of time of onset of each intensity of seizure and tallying of maximal seizure intensity in 2-min bins. Seizure behavior typically subsides by 30 min after PTZ administration, so animals were then returned to their home cages. Animals were killed, brains collected, and sections examined by epifluorescence microscopy to confirm appropriate transduction of hippocampal CA1.

### Experimental design and statistical analysis

All image analysis was performed using ImageJ (RRID:SCR_003070). For analysis of immunofluorescence, brain sections were analyzed from mice injected with HACB2-AAV (VGLUT1-cre: *n* = 6, VGAT-cre: *n* = 4) or EGFP-AAV (VGLUT1-cre: *n* = 6, VGAT-cre: *n* = 4). For each animal, each quantified value is the average of 10–15 measurements [regions of interest (ROIs) or individual interneurons], as explained below. Because of the densely packed nature of transduced CA1 pyramidal cells in VGLUT1-cre mice, ROIs were used to measure impact of viral transduction. In each section, five images were taken per hemisphere, and three ROIs with areas of 500 μm^2^ were created within stratum pyramidale (SP) of CA1 in each to determine changes in perisomatic synapses. These values were averaged to present a single value for each animal. This was repeated within stratum radiatum (SR) and stratum oriens (SO) to measure changes in dendritic synapses. To determine changes in synaptic proteins in transduced interneurons of CA1 (of VGAT-cre mice), cell bodies within SP were outlined and then expanded inward/outward to create a 15-pixel thick ring-shaped ROI around transduced neurons. VGAT, gephyrin, and GABA_A_R-γ2-subunit immunoreactivities were then measured in these ROIs. Five to seven cells were measured per brain section, two sections per animal, to calculate an average value for each animal. Since only one hemisphere was injected, the other hemisphere served as an internal control to ensure there was no significant variability from mouse to mouse. Additional injected mice were excluded from study in cases of off-target injection or lack of transduction (VGAT-cre: four mice, VGLUT1-cre: three mice). For electrophysiological and behavioral study in VGLUT1-cre mice, mice were injected bilaterally. For electrophysiological experiments, data are presented from transduced cells (HACB2-AAV: *n* = 5, EGFP-AAV: *n* = 6; 1 cell per mouse) from injected mice. The final 100 events before bath application of picrotoxin was used for analysis. For behavioral experiments, data are presented for injected mice (HACB2-AAV: *n* = 15, EGFP-AAV: *n* = 12), and the observer was blinded to the condition of the mouse during seizure scoring. Additional injected mice were excluded from study if they died after PTZ administration (HACB2-AAV: one mouse, EGFP-AAV: two mice) or if brain examination determined off-target injection or lack of transduction. No additional mortality was observed during or following procedures in adeno-associated virus (AAV)-injected mice. Statistical analyses were performed using Prism 8 (GraphPad). Statistical tests include Mann–Whitney *U* tests for two-group comparisons when sample size was below 10, and unpaired *t* tests when sample size was >10, Kolmogorov–Smirnov tests, and Fisher’s exact test. Statistical data can be found in Table 1. Summary data are presented as mean ± SEM from *n* mice or neurons, as indicated.

**Table 1:** Descriptive and summary statistics by figure

Data	Figure	Statistical test	Test values	*p* value
Immunofluorescence data:				
*VGLUT1-cre:*				
HA MFI	1	Mann–Whitney *U* test	Median EGFP: 22.09 Median CB: 57.90 *N* = 6, *U* = 0	0.0022
CB integrated FI	1	Mann–Whitney *U* test	Median EGFP: 283.3 Median CB: 384.7 *N* = 6, *U* = 5	0.0411
SO Geph size	2	Mann–Whitney *U* test	Median EGFP: 0.2559 Median CB: 0.3428 *N* = 6, *U* = 0	0.0022
SP Geph size	2	Mann–Whitney *U* test	Median EGFP: 0.1923 Median CB: 0.2491 *N* = 6, *U* = 2	0.0087
SR Geph size	2	Mann–Whitney *U* test	Median EGFP: 0.2360 Median CB: 0.2838 *N* = 6, *U* = 4	0.0260
SO Geph density	2	Mann–Whitney *U* test	Median EGFP: 0.1133 Median CB: 0.1691 *N* = 6, *U* = 1	0.0043
SP Geph density	2	Mann–Whitney *U* test	Median EGFP: 0.0723 Median CB: 0.1331 *N* = 6, *U* = 3	0.0152
SR Geph density	2	Mann–Whitney *U* test	Median EGFP: 0.1136 Median CB: 0.1922 *N* = 6, *U* = 3	0.0152
SO γ2 size	2	Mann–Whitney *U* test	Median EGFP: 0.1760 Median CB: 0.1715 *N* = 6, *U* = 11	0.8413
SP γ2 size	2	Mann–Whitney *U* test	Median EGFP: 0.1647 Median CB: 0.2013 *N* = 6, *U* = 2	0.0317
SR γ2 size	2	Mann–Whitney *U* test	Median EGFP: 0.1522 Median CB: 0.1562 *N* = 6, *U* = 10	0.6905
SO γ2 density	2	Mann–Whitney *U* test	Median EGFP: 0.0879 Median CB: 0.0975 *N* = 6, *U* = 12	>0.9999
SP γ2 density	2	Mann–Whitney *U* test	Median EGFP: 0.0865 Median CB: 0.0824 *N* = 6, *U* = 12	>0.9999
SR γ2 density	2	Mann–Whitney *U* test	Median EGFP: 0.0615 Median CB: 0.0877 *N* = 6, *U* = 6	0.2222
SO VGAT size	2	Mann–Whitney *U* test	Median EGFP: 0.2082 Median CB: 0.2406 *N* = 6, *U* = 9	0.1797
SP VGAT size	2	Mann–Whitney *U* test	Median EGFP: 0.2447 Median CB: 0.2910 *N* = 6, *U* = 10	0.2403
SR VGAT size	2	Mann–Whitney *U* test	Median EGFP: 0.2015 Median CB: 0.2072 *N* = 6, *U* = 12	0.3939
SO VGAT density	2	Mann–Whitney *U* test	Median EGFP: 0.0594 Median CB: 0.0716 *N* = 6, *U* = 10	0.2229
SP VGAT density	2	Mann–Whitney *U* test	Median EGFP: 0.0705 Median CB: 0.0799 *N* = 6, *U* = 7	0.0931
SR VGAT density	2	Mann–Whitney *U* test	Median EGFP: 0.0778 Median CB: 0.0787 *N* = 6, *U* = 16	0.8182
*VGAT-cre*				
HA MFI	1	Mann–Whitney *U* test	Median EGFP: 25.93 Median CB: 128.9 *N* = 4, *U* = 0	0.0286
CB integrated FI	1	Mann–Whitney *U* test	Median EGFP: 450.8 Median CB: 580.9 *N* = 4, *U* = 0	0.0286
Geph size	3	Mann–Whitney *U* test	Median EGFP: 0.1788 Median CB: 0.3437 *N* = 4, *U* = 0	0.0286
Geph density	3	Mann–Whitney *U* test	Median EGFP: 0.2291 Median CB: 0.4403 *N* = 4, *U* = 0	0.0286
γ2 size	3	Mann–Whitney *U* test	Median EGFP: 0.1706 Median CB: 0.2151 *N* = 4, *U* = 1	0.0571
γ2 density	3	Mann–Whitney *U* test	Median EGFP: 0.1387 Median CB: 0.1386 *N* = 4, *U* = 8	>0.9999
VGAT size	3	Mann–Whitney *U* test	Median EGFP: 0.3144 Median CB: 0.3617 *N* = 4, *U* = 4	0.3429
VGAT density	3	Mann–Whitney *U* test	Median EGFP: 0.2008 Median CB: 0.2967 *N* = 4, *U* = 5	0.4857
Electrophysiology data				
mIPSC amplitude cumulative frequency	4	Kolmogorov–Smirnov test	Kolmogorov–Smirnov *D* = 0.3283 *N* = 600, 500	<0.0001
mIPSC amplitude	4	Mann–Whitney *U* test	Median EGFP: −29.84 Median CB: −41.98 *N* = 6, 5, *U* = 4	0.0455
mIPSC frequency cumulative frequency	4	Kolmogorov–Smirnov test	Kolmogorov–Smirnov *D* = 0.1651 *N* = 600, 500	<0.0001
mIPSC frequency	4	Mann–Whitney *U* test	Median EGFP: 0.2125 Median CB: 0.4583 *N* = 6, 5, *U* = 0	0.0043
mIPSC rise time	4	Mann–Whitney *U* test	Median EGFP: 0.9404 Median CB: 1.127 *N* = 6, 5, *U* = 0	0.0043
mIPSC decay Tau	4	Mann–Whitney *U* test	Median EGFP: 15.22 Median CB: 18.40 *N* = 6, 5, *U* = 6	0.1255
Behavioral data				
Cumulative seizure score	5	Kolmogorov–Smirnov test	Kolmogorov–Smirnov *D* = 0.7333 *N* = 12, 15	0.0006
Latency to seizure score 1	5	Unpaired *t* test	Mean EGFP: 76.42 Mean CB: 216.1 *N* = 12, 14, *F* = 55.84, DFn = 13, Dfd = 11	0.0358
Latency to seizure score 2	5	Unpaired *t* test	Mean EGFP: 83.33 Mean CB: 260.0 *N* = 12, 14, *F* = 90.32, DFn = 13, Dfd = 11	0.0480
Latency to seizure score 3	5	Unpaired *t* test	Mean EGFP: 108.4 Mean CB: 252.5 *N* = 12, 11, *F* = 45.64, DFn = 10, Dfd = 11	0.0912
Latency to seizure score 4	5	Unpaired *t* test	Mean EGFP: 167.6 Mean CB: 269.9 *N* = 12, 9, *F* = 5.961, DFn = 8, Dfd = 11	0.1815
Latency to seizure score 5	5	Unpaired *t* test	Mean EGFP: 210.5 Mean CB: 381.0 *N* = 10, 5, *F* = 10.95, DFn = 4, Dfd = 9	0.1062
Latency to seizure score 6	5	Unpaired *t* test	Mean EGFP: 239.9 Mean CB: 298.0 *N* = 9, 2, *F* = n/a, DFn = n/a, Dfd = n/a	0.5353
Contingency seizure score 1	5	Fisher’s exact test	EGFP: 12, 0 CB: 14, 1 Total: 26, 1 (seized, did not)	>0.9999
Contingency Seizure score 2	5	Fisher’s exact test	EGFP: 12, 0 CB: 14, 1 Total: 26, 1 (seized, did not)	>0.9999
Contingency seizure score 3	5	Fisher’s exact test	EGFP: 12, 0 CB: 11, 4 Total: 23, 4 (seized, did not)	0.1060
Contingency seizure score 4	5	Fisher’s exact test	EGFP: 12, 0 CB: 9, 6 Total: 21, 6 (seized, did not)	0.0200
Contingency seizure score 5	5	Fisher’s exact test	EGFP: 10, 5 CB: 5, 10 Total: 15, 12 (seized, did not)	0.0185
Contingency seizure score 6	5	Fisher’s exact test	EGFP: 9, 3 CB: 2, 13 Total: 11, 16 (seized, did not)	0.0020

## Results

### Mice injected with AAV-HA-CB2SH3– express both mCitrine and HA-CB

In order to validate that our cre-dependent viral construct was capable of transduction of appropriate cells in CA1, we examined the pattern of mCitrine and HA expression in injected mice.

In VGLUT1-IRES-cre mice injected with the HACB2-AAV, large swathes of the pyramidal cell layer in CA1 expressed mCitrine (Fig. 1*A*), interspersed with the occasional non-transduced cell, likely indicating an interneuron. As expected, none of the cells in SO were transduced, since cell bodies there belong to GABAergic cells. The transduced cells showing mCitrine fluorescence also expressed HA-CB2SH3–, as detected by immunoreactivity with Ms anti-HA (Fig. 1*B*) and increased immunoreactivity with Rb anti-CB (Fig. 1*D*). In CA1 of the VGLUT1-IRES-cre mice injected with the EGFP-AAV, we did not detect any HA (Fig. 1*C*), nor any increased levels of CB (Fig. 1*E*). To quantify the extent to which CB was expressed over endogenous levels, confocal images were taken (Fig. 1*F*,*G*). Mean immunofluorescence intensity (MFI) of HA was significantly increased in HACB2-AAV-transduced neurons (CB: 71.4 ± 10.3 A.U.; EGFP: 20.8 ± 2.8 A.U.; Mann–Whitney *U* test, *p* = 0.002; Fig. 1*H*). Immunofluorescence with the rabbit anti-CB antibody showed that while there was no significant change in the size of CB clusters (Mann–Whitney *U* test, *p* = 0.49), there were increases in the density (CB: 0.265 ± 0.017 clusters/μm^2^; EGFP: 0.227 ± 0.021 clusters/μm^2^; Mann–Whitney *U* test, *p* = 0.041) and integrated fluorescence intensity of the immunopositive puncta (CB: 386.2 ± 35.4 A.U.; EGFP: 287.4 ± 14.6 A.U.; Mann–Whitney *U* test, *p* = 0.041; Fig. 1*I*).

In injected VGAT-IRES-cre mice, mCitrine expression in dorsal hippocampal CA1 matched the expected distribution of interneurons (Fig. 1*J*), with transduced cells scattered through pyramidal cell layer, as well as cell bodies in the SO. Since our viral construct was inducing bicistronic expression of mCitrine and HA-CB2SH3–, we confirmed that transduced cells were also expressing HA-CB2SH3–. We were able to detect both HA (Fig. 1*K*) and an increased level of CB (Fig. 1*M*) in transduced cells of CA1 in mice injected with the HACB2-AAV compared with EGFP-AAV-injected mice (Fig. 1*L*,*N*, respectively). As expected, all neurons in the SP of EGFP-AAV-injected mice showed endogenous CB expression. However, in HACB2-AAV transduced interneurons, CB expression was noticeably stronger than the endogenous CB expression of either the surrounding non-transduced pyramidal neurons (Fig. 1*M*) or interneurons transduced with EGFP-AAV (Fig. 1*M* vs *N*,*O* vs *P*). Analysis of confocal images (Fig. 1*O*,*P*) revealed that HA MFI is significantly increased in HACB2-AAV transduced interneurons (CB: 133.3 ± 11.7 A.U.; EGFP: 25.3 ± 1.1 A.U.; Mann–Whitney *U* test, *p* = 0.029; Fig. 1*Q*) as is integrated fluorescence intensity of CB puncta (CB: 617.6 ± 59.5; EGFP: 428.3 ± 42.6 A.U.; Mann–Whitney *U* test, *p* = 0.029; Fig. 1*R*). While there was no change in the density of the clusters (Mann–Whitney *U* test, *p* = 0.69), there was a moderate but not significant increase in size of clusters (CB: 0.211 ± 0.019 μm^2^; EGFP: 0.161 ± 0.008 μm^2^ Mann–Whitney *U* test, *p* = 0.057).

### Conditional overexpression of HA-CB2SH3– in CA1 pyramidal neurons results in enhanced gephyrin and perisomatic GABAAR- γ2 clusters in VGLUT1-cre mice

**Figure 2.**
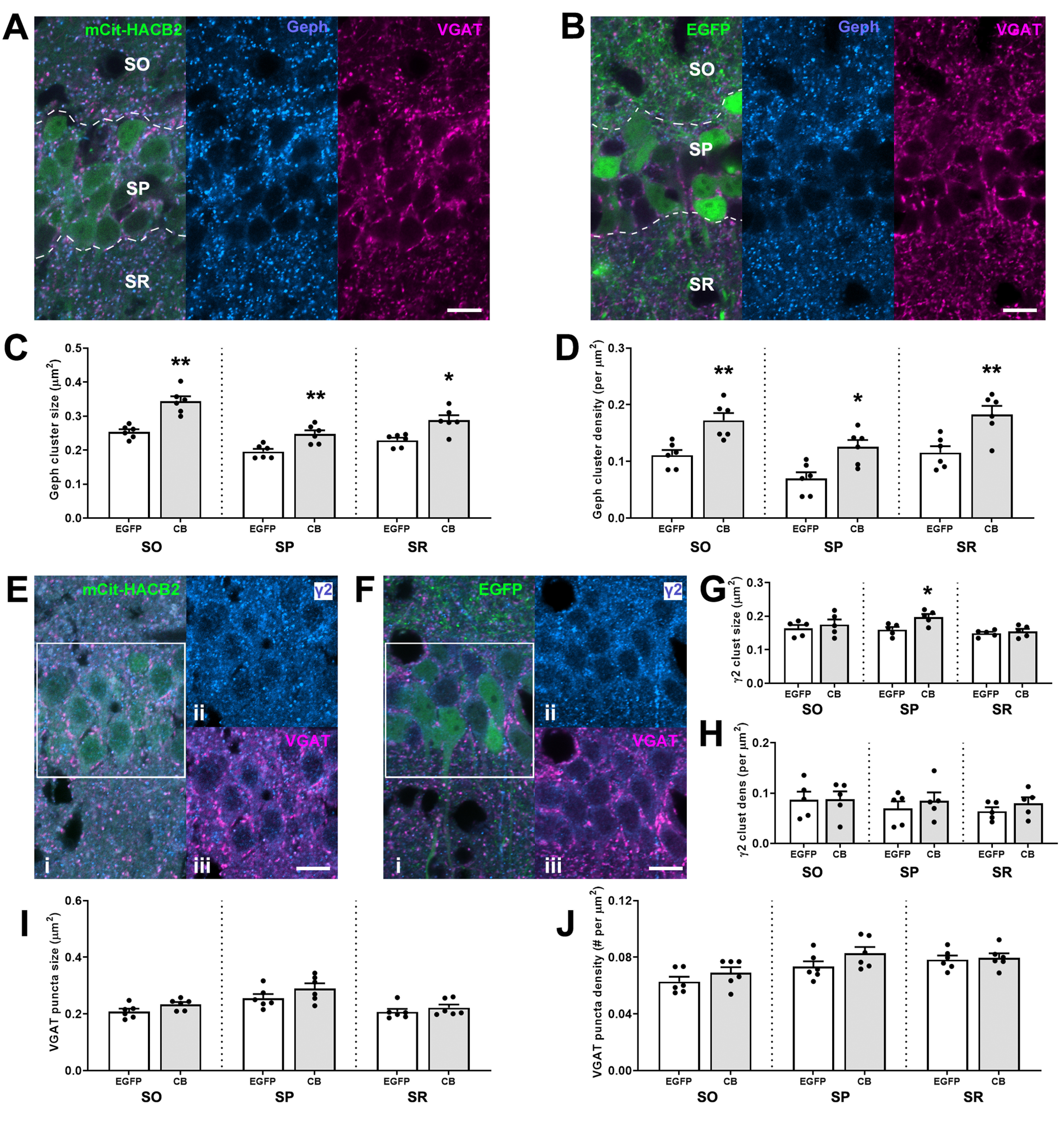
Overexpression of HA-CB2SH3– in hippocampal CA1 pyramidal cells of the VGLUT1-IRES-cre mouse results in larger postsynaptic gephyrin clusters without observed alteration of the presynaptic GABAergic input. ***A***, ***B***, Representative IF images of CA1 pyramidal cells transduced with mCitrine-HACB2-AAV (***A***) or EGFP-AAV (***B***) labeled with Ms anti-Geph (blue) and GP anti-VGAT (magenta). Outlined in ***A***, ***B*** are rough borders for SO, SP, and SR. ***C***, ***D***, Quantification of size (***C***) and density (***D***) of gephyrin clusters in these regions. ***E***, ***F***, Representative IF images of CA1 pyramidal cells transduced with mCitrine-HACB2-AAV (***E***) or EGFP-AAV (***F***) labeled with Ms anti- γ2 (blue; ***i***, ***ii***) and GP anti-VGAT (magenta; ***i***, ***iii***). Panels ***ii***, ***iii*** show individual channels for blue and red for SP as outlined in the box in ***i***. ***G***, ***H***, Quantification of size (***G***) and density (***H***) of GABA_A_R γ2 clusters in these regions. ***I***, ***J***, Quantification of size (***I***) and density (***J***) of VGAT puncta in these regions. Each dot represents the mean for quantification for one animal, and error bars indicate SEM. At least two sections were processed per animal, with three ROIs in each region (SO, SP, SR) for each of five images for each hemisphere (total sampled area per region/hemisphere = 7500 μm^2^). Scale bar: 10 μm. Asterisks in the quantification graphs indicate significance using Mann–Whitney *U* test; *p < 0.05, **p < 0.01.

To investigate the extent to which overexpression of HA-CB2SH3– affects hippocampal GABAergic synapses, we examined presynaptic and postsynaptic protein expression in HACB2-AAV transduced glutamatergic pyramidal cells of CA1 (Fig. 2*A*), as compared with those transduced with EGFP-AAV (Fig. 2*B*). Because of the packed nature of the transduced hippocampal pyramidal cells, regions within CA1 were examined rather than individual transduced cells: ROIs within the SP that contain the cell bodies of the pyramidal cells, or ROIs within SO or SR populated by processes of the pyramidal cells. In VGLUT1-cre mice injected with the HACB2-AAV, there were significant increases in average size of gephyrin clusters in each region of the transduced CA1 (SO: CB: 0.343 ± 0.015 μm^2^, EGFP: 0.253 ± 0.008 μm^2^; SP: CB: 0.247 ± 0.011 μm^2^, EGFP: 0.195 ± 0.008 μm^2^; SR: CB: 0.288 ± 0.014 μm^2^, EGFP: 0.229 ± 0.008 μm^2^; Mann–Whitney *U* test, *p* = 0.002, *p* = 0.009, *p* = 0.026; respectively; Fig. 2*C*). The gephyrin clusters in these regions of HACB2-AAV-transduced CA1 also had increased MFI in the SO (32.4% increase), SP (61.5% increase), and SR (43.7% increase) compared with EGFP-AAV-transduced CA1 (Mann–Whitney *U* test, *p* = 0.026, *p* = 0.004, and *p* = 0.009, respectively). There were also more clusters in these regions of transduced CA1 (SO: CB: 0.172 ± 0.013 per μm^2^, EGFP: 0.111 ± 0.009 per μm^2^; SP: CB: 0.126 ± 0.012 per μm^2^, EGFP: 0.070 ± 0.11 per μm^2^; SR: CB: 0.183 ± 0.015 per μm^2^, EGFP: 0.116 ± 0.011 per μm^2^; Mann–Whitney *U* test, *p* = 0.004, *p* = 0.015, and *p* = 0.015; respectively; Fig. 2*D*). Size, density and MFI of gephyrin clusters were also increased compared with non-transduced contralateral hemisphere in the HACB2-AAV-injected mouse brains. There were no significant differences in any of the measured parameters between contralateral hemispheres of HACB2-AAV-injected mice and EGFP-AAV-injected mice.

In the SP of the HACB2-AAV-injected mice, there was also an increase of size of γ2 GABA_A_R subunit clusters compared with the EGFP-AAV-injected mice (CB: 0.197 ± 0.009 μm^2^ vs EGFP: 0.160 ± 0.008 μm^2^; Mann–Whitney *U* test, *p* = 0.032; Fig. 2*E–H*). No significant differences in GABA_A_R γ2 density or MFI were detected in the SP. Moreover, no difference in cluster size, density or MFI were found in the SO or SR of HACB2-AAV versus EGFP-AAV-injected mice.

Regarding presynaptic VGAT expression, there was no significant change to puncta size, density, or MFI of the presynaptic VGAT in SO, SP or SR in the HACB2-AAV versus EGFP-AAV-injected mice (Fig. 2*I*,*J*).

### Conditional overexpression of HA-CB2SH3– enhances gephyrin clusters in CA1 interneurons in VGAT-cre mice

**Figure 3.**
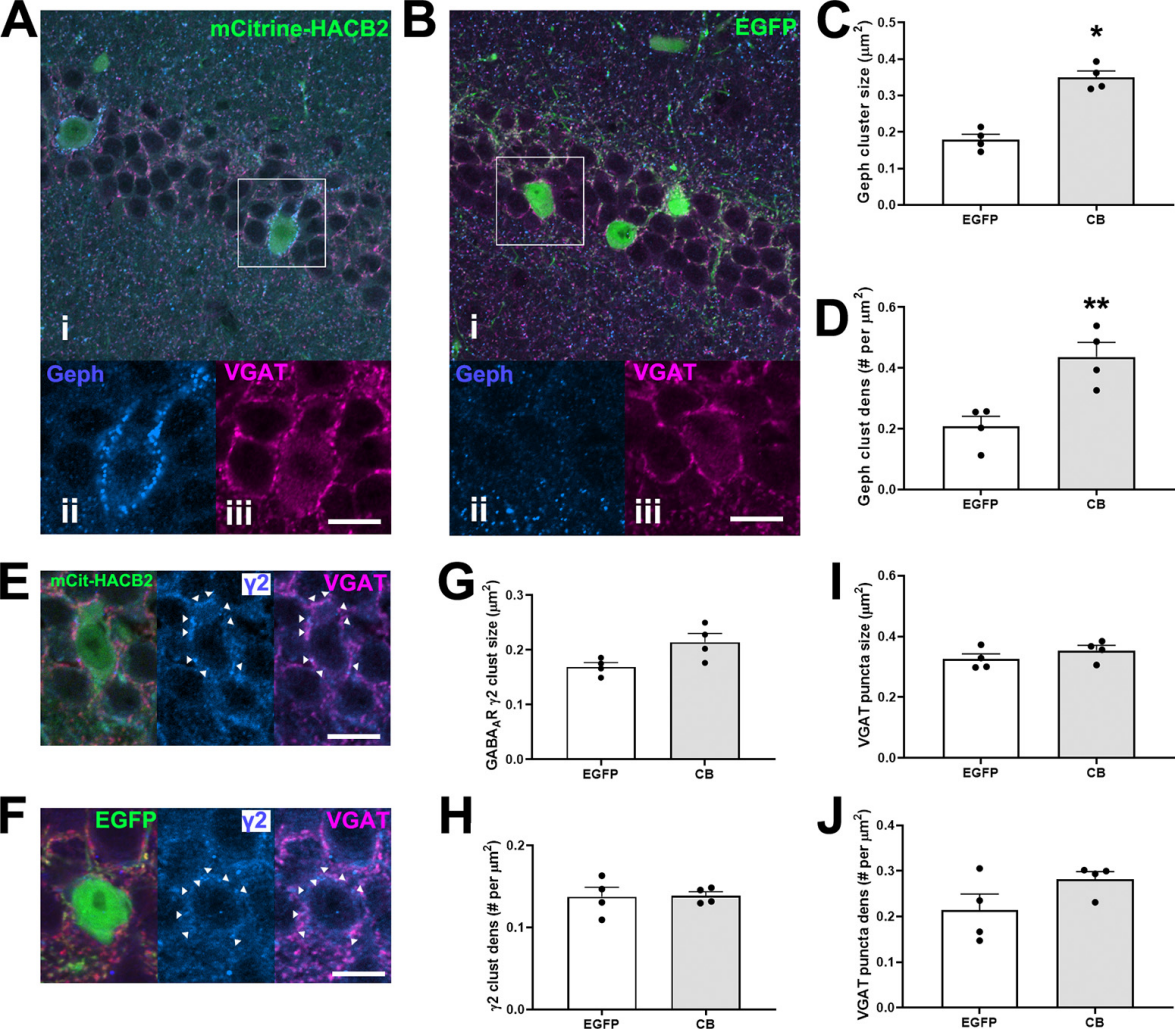
Overexpression of HA-CB2SH3– in hippocampal interneurons of the VGAT-IRES-cre mouse results in larger gephyrin clusters without observed alterations of presynaptic VGAT input. ***A***, ***B***, Representative IF images of CA1 interneurons transduced with mCitrine-HACB2-AAV (***A***) or EGFP-AAV (***B***) labeled with Ms anti-Geph (blue; ***ii***) and GP anti-VGAT (magenta; ***iii***). Inset of boxes in ***i*** are shown in ***ii***, ***iii***. ***C***, ***D***, Quantification of the size (***C***) and density (***D***) of the gephyrin clusters on the perimeter of the transduced cells. ***E***, ***F***, Representative IF images of CA1 interneurons transduced with mCitrine-HACB2-AAV (***E***) or EGFP-AAV (***F***) labeled with Rb anti-γ2 (blue; ***ii***) and GP anti-VGAT (magenta; ***iii***). ***G***, ***H***, The size (***G***) and density (***H***) of the GABA_A_R γ2 clusters on the perimeter of the transduced cells were quantified. The size (***I***) and density (***J***) of the VGAT puncta surrounding the transduced cell have been quantified. Each dot represents one animal and error bars indicate SEM. Two sections per animal were processed and at least five cells per brain section were quantified. Scale bars: 20 μm (***A***, ***Bi***) and 10 μm (***A***, ***Bii***, ***Biii***, ***E***, ***F***). Asterisks in the quantification graphs indicate significance using Mann–Whitney *U* test; *p < 0.05, **p < 0.01.

We examined the changes to presynaptic and postsynaptic proteins at the GABAergic synapse as a result of overexpression of HA-CB2SH3– in CA1 interneurons (Fig. 3*A*). There was a marked increase in the size of perisomatic gephyrin clusters on neurons transduced with the HACB2-AAV (0.350 ± 0.017 μm^2^) compared with those transduced with the EGFP-AAV (0.179 ± 0.015 μm^2^; Mann–Whitney *U* test, *p* = 0.029; Fig. 3*A–C*) and a 140% increase in MFI of clusters (Mann–Whitney *U* test, *p* = 0.029). This was accompanied by an increase in the density of gephyrin clusters in neurons transduced with HACB2-AAV compared with EGFP-AAV (0.436 ± 0.047 and 0.207 ± 0.034 clusters/μm^2^, respectively; Mann–Whitney *U* test, *p* = 0.029; Fig. 3*D*). Despite gephyrin clusters in HACB2-AAV transduced interneurons being nearly twice the size, and twice as numerous as in EGFP-AAV transduced interneurons, there was only a modest, but not significant, increase in the size of GABA_A_R γ2 clusters (CB: 0.214 ± 0.016 vs EGFP: 0.169 ± 0.008 μm^2^; Mann–Whitney *U* test, *p* = 0.057; Fig. 3*E–G*) and no significant change in density of clusters (CB: 0.139 ± 0.005 vs EGFP: 0.138 ± 0.011 puncta/μm^2^; Mann–Whitney *U* test, *p* > 0.99; Fig. 3*H*).

There were no corresponding changes in either size (CB: 0.353 ± 0.017 μm^2^; EGFP: 0.325 ± 0.017 μm^2^; Mann–Whitney *U* test, *p* = 0.34; Fig. 3*I*) or density (CB: 0.282 ± 0.017 puncta/μm^2^; EGFP: 0.214 ± 0.036 puncta/μm^2^; Mann–Whitney *U* test, *p* = 0.49; Fig. 3*J*) in VGAT-positive terminals synapsing onto transduced cells. Additionally, there were no differences in overall gephyrin, GABA_A_R γ2, or VGAT cluster size or density in the contralateral CA1 between HACB2-AAV-injected and EGFP-AAV-injected mice.

### Synaptic inhibitory neurotransmission is strengthened in HA-CB2SH3– overexpressing CA1 pyramidal neurons

**Figure 4.**
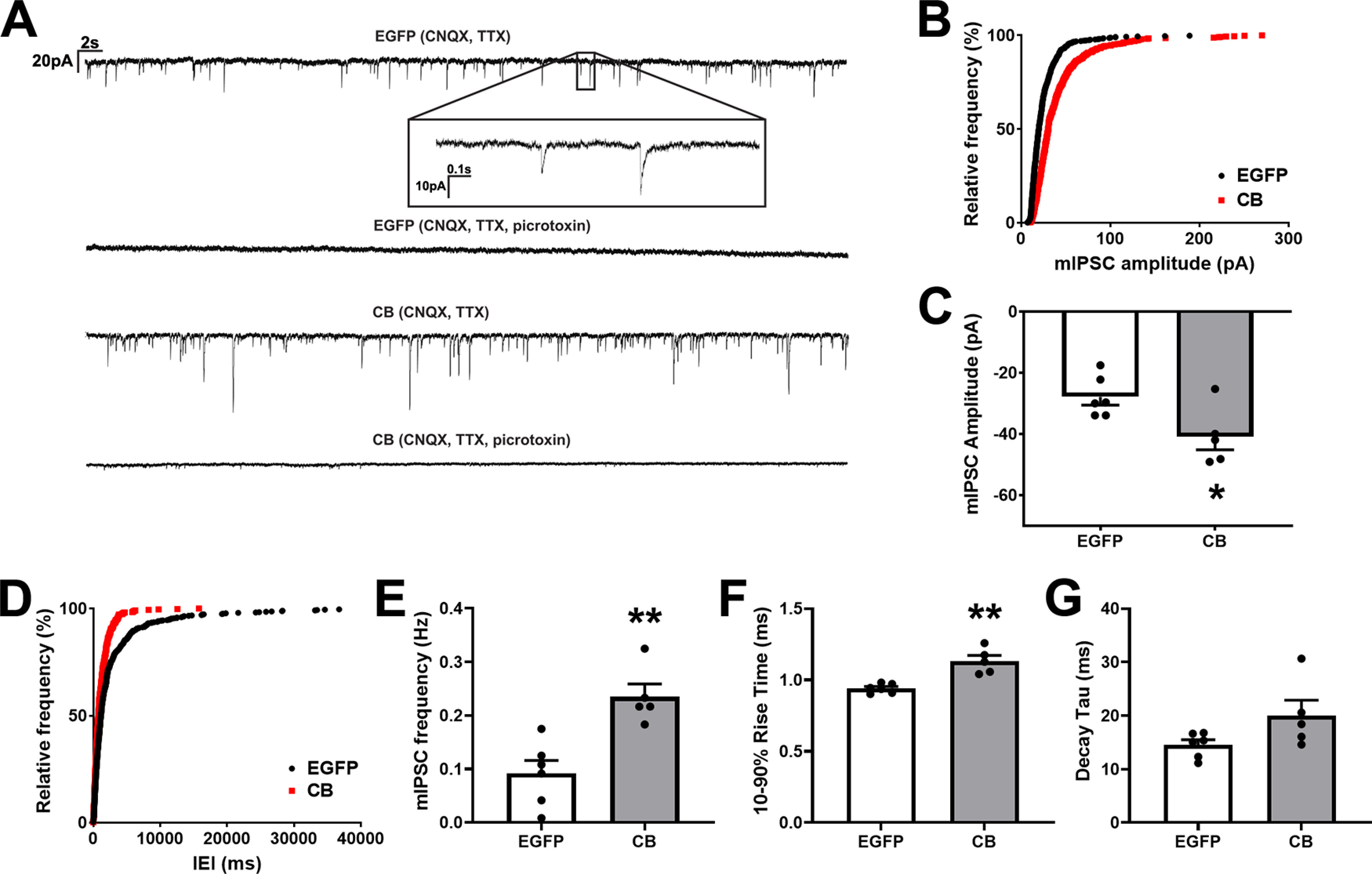
Pyramidal CA1 cells of the VGLUT1-IRES-cre mouse transduced with HACB2-AAV have increased strength of synaptic inhibitory neurotransmission. ***A***, Representative traces showing mIPSCs from EGFP-AAV-transduced cells (top) or mCitrine-HACB2-AAV-transduced cells (bottom) that were eliminated in the presence of picrotoxin (lower trace for each). Inset of upper trace shows stereotypical mIPSCs. Vertical scale bar indicates 20 pA and horizontal bar indicates 2 s. Inset, Vertical scale bar indicates 10 pA; while horizontal bar indicates 0.1 s. ***B***, Amplitudes of mIPSCs were quantified and the cumulative relative frequency displayed. ***C***, The average amplitudes of mIPSCs. ***D***, Interevent interval (IEI) of mIPSCs were quantified and the cumulative relative frequency displayed. ***E***, Quantification of the average frequency of mIPSCs. ***F***, ***G***, Quantification of the average 10–90% rise time (***F***), and decay tau (***G***). For ***C***, ***E–G***, each dot represents one cell from which 100 events were quantified, and error bars indicate SEM. For ***B***, ***D***, Kolmogorov–Smirnov tests were performed; *p* < 0.001. Asterisks indicate significance using Mann–Whitney *U* test; *p < 0.05, **p < 0.01.

To determine whether the enlarged gephyrin and GABA_A_R clusters in VGLUT1-cre mice transduced with HACB2-AAV corresponded to stronger synaptic inhibitory neurotransmission, we measured spontaneous mIPSCs in transduced CA1 pyramidal neurons in brain slices from mice bilaterally injected with either HACB2-AAV or control EGFP-AAV. We found that in HACB2-AAV transduced CA1 pyramidal cells, there were more inhibitory events, many with higher amplitudes than in the control EGFP-AAV-transduced cells (Fig. 4*A*). These mIPSCs were eliminated in the presence of picrotoxin, ascertaining that they correspond to GABA_A_R currents.

A cumulative relative frequency plot indicates a right shift in the amplitudes of mIPSCs in HACB2-AAV-transduced neurons (Kolmogorov–Smirnov *p* < 0.001; Fig. 4*B*) and greater amplitudes (CB: −40.91 ± 4.28 pA; EGFP: −27.88 ± 2.71 pA; Mann–Whitney *U* test, *p* = 0.046; Fig. 4*C*), suggesting an increase in the number of postsynaptic GABA_A_Rs.

The mIPSCs in HACB2-AAV-transduced cells shifted to lower interevent intervals (Kolmogorov–Smirnov, *p* < 0.001; Fig. 4*D*) and these transduced neurons demonstrated higher frequencies (CB: 0.537 ± 0.095 Hz; EGFP: 0.181 ± 0.036 Hz; Mann–Whitney *U* test, *p* = 0.004; Fig. 4*E*) than control EGFP-AAV cells. This finding when taken together with our results demonstrating no change in size or density of presynaptic VGAT, likely indicates a higher probability of release from presynaptic GABAergic terminals. Finally, we analyzed the rise time and decay tau of mIPSCs, to determine whether there might be any changes in receptor kinetics which might speak to alterations in distribution of receptors or receptor subunit composition. We found that while there was a significant increase in 10–90% rise times of mIPSCs in HACB2-AAV neurons (CB: 1.132 ± 0.040 ms; EGFP: 0.941 ± 0.013 ms; Mann–Whitney *U* test, *p* = 0.004; Fig. 4*F*), the increase in decay tau was not significant (CB: 20.05 ± 2.84 ms; EGFP: 14.55 ± 0.95 ms; Mann–Whitney *U* test, *p* = 0.126; Fig. 4*G*).

### VGLUT1-cre mice injected with HACB2-AAV are less susceptible to acute seizures

**Figure 5.**
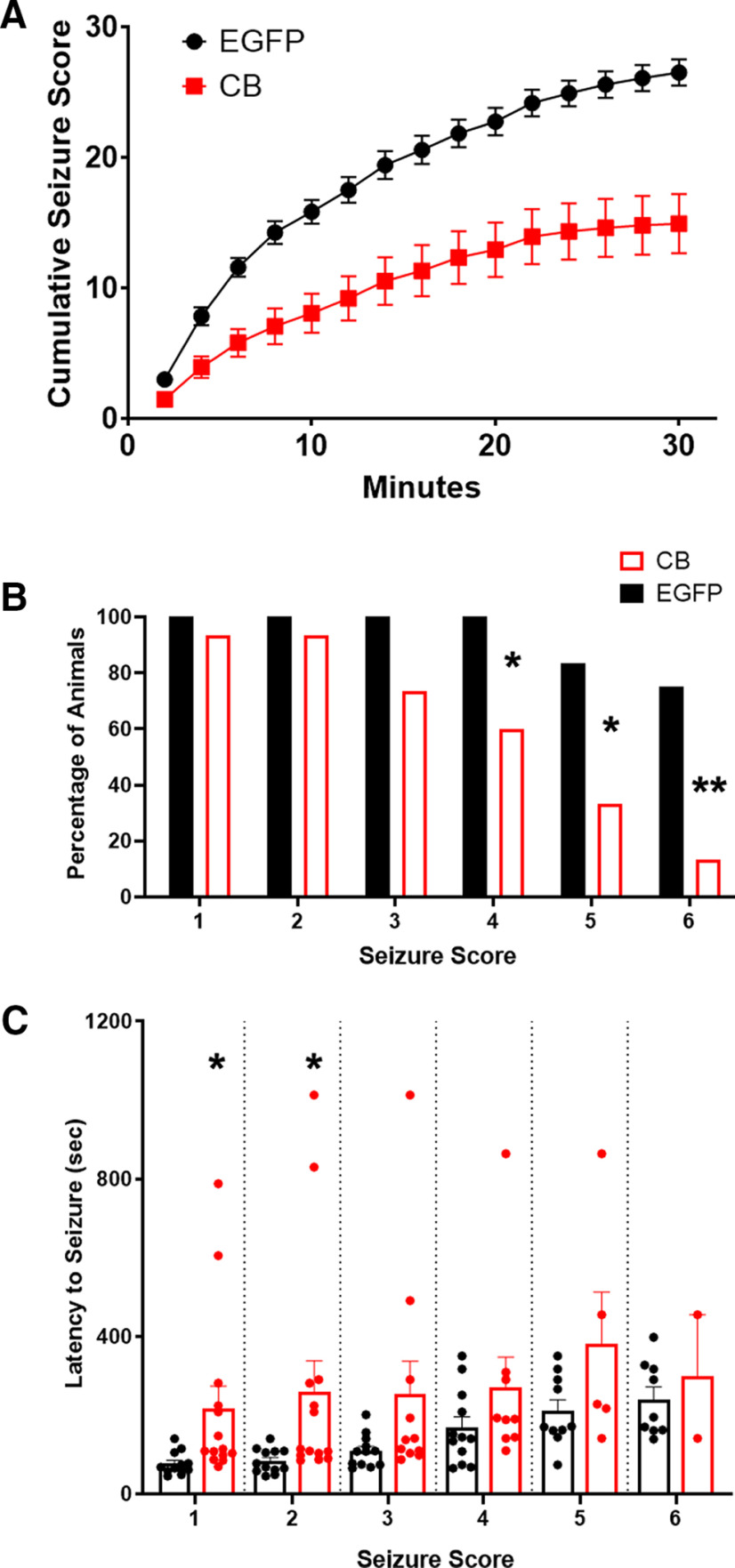
VGLUT1-IRES-cre mouse injected intrahippocampally with mCitrine-HACB2-AAV display lower susceptibility to PTZ-induced intense seizures. ***A***, Seizures were scored along a modified Racine scale and a maximal seizure score assigned in 2-min bins, and average cumulative scores are presented. ***B***, The percentage of animals that had a seizure at each intensity score. Asterisks indicate significance using Fisher’s exact test; *p < 0.05, **p < 0.01. ***C***, The average latency to the first seizure of each intensity was recorded. Error bars indicate SEM, and each dot represents one animal. Note that at higher intensity seizure scores, there are fewer mice that seize, particularly in the CB category. Most mice seize with lower intensities. Asterisks indicate significance using unpaired *t* test; *p < 0.05.

To determine whether increased GABAergic inhibitory synaptic neurotransmission in CA1 pyramidal cells, seen in VGLUT1-cre mice injected with the HACB2-AAV, imparted neuroprotective effects against acute seizures, we subjected AAV-injected mice to intraperitoneal injections of PTZ. Based on initial studies we settled on a dose of PTZ (40 mg/kg), which induced seizures reliably in naive animals with minimal mortality. Three weeks after bilateral CA1 injection with HACB2-AAV (*n* = 15 mice) or a control EGFP-AAV (*n* = 12 mice), we assessed seizures along a modified Racine scale after a single dose of PTZ. The mice injected with the HACB2-AAV displayed lower cumulative seizure scores over the 30 min observed, compared with the EGFP-AAV control mice (Kolmogorov–Smirnov test, *p* < 0.001; Fig. 5*A*). HACB2-AAV-injected mice were less likely to have higher intensity seizures after PTZ compared with the control mice (Fisher’s exact, *p* = 0.020, *p* = 0.019, and *p* = 0.002; seizure scores 4, 5, and 6, respectively; Fig. 5*B*). When comparing the onset latency to the first seizure of each intensity score (Fig. 5*C*), we found an increased latency period for the HACB2-AAV mice for seizure scores 1 and 2 (CB: 216.1 ± 16.6s, EGFP: 76.4 ± 2.6s; and CB: 260.0 ± 22.4s, EGFP: 83.3 ± 2.8s; unpaired *t* test, *p* = 0.031 and *p* = 0.042, respectively). However, the increased latency times were not statistically significant at the higher seizure scores because of the low numbers of HACB2-AAV mice having high intensity seizures (note the diminishing number of data points in Fig. 5*C*).

## Discussion

We evaluated the effects of overexpressing CB in specific neuron types in the adult dorsal hippocampus. While CBSH3+ splice variants are the far more abundantly expressed isoforms of CB (Harvey et al., 2004), they are autoinhibited in their ability to induce clustering of gephyrin, unless relieved by other postsynaptic proteins (Poulopoulos et al., 2009; Mayer et al., 2013; Soykan et al., 2014). The constitutively active CBSH3– isoforms, however, are capable of inducing the accumulation of gephyrin and GABA_A_Rs at postsynaptic sites (Chiou et al., 2011; Tyagarajan et al., 2011; Fekete et al., 2017). We found that overexpression of CB2SH3– in hippocampal CA1 pyramidal neurons resulted in significantly increased CB, gephyrin and GABA_A_R clustering in perisomatic regions (within SP). Overexpression of CB2SH3– in CA1 interneurons also resulted in increased CB expression, and larger perisomatic gephyrin and GABA_A_R clusters. The increase in postsynaptic GABAergic receptors was accompanied by a significantly increased mIPSC amplitude and frequency. Overexpression of CB2SH3– in excitatory neurons in CA1 of the dorsal hippocampus also provided resilience against PTZ-induced seizures.

In CA1 pyramidal cells, overexpression of CB2SH3– resulted in an increase in gephyrin and GABA_A_R cluster size. The majority of these large gephyrin clusters were apposed to VGAT terminals, indicating localization to GABAergic synapses. This is consistent with what has been seen in cultured hippocampal neurons transfected to overexpress CB2SH3– (Chiou et al., 2011; Tyagarajan et al., 2011; Körber et al., 2012) and in CB2SH3– transfected pyramidal neurons of the cerebral cortex of *in utero* electroporated (IUE) rats (Fekete et al., 2017). In HACB2-AAV transduced hippocampal pyramidal cells, the increase in amplitude of spontaneous mIPSCs further indicates an accumulation of GABA_A_Rs at the postsynapse.

Immunofluorescence revealed an increase in synaptic γ2-containing GABA_A_Rs in SP, but not in SO or SR, despite CA1 pyramidal cells receiving 92% of their GABAergic synaptic input at their dendrites (Megías et al., 2001). Each cell compartment is typically innervated by different interneuron subtypes, with parvalbumin (PV)+ and cholecystokinin (CCK)+ basket cells providing the majority of GABAergic innervation to perisomatic regions (Miles et al., 1996; Salesse et al., 2011). In addition, PV+ chandelier cells almost exclusively innervate the axon initial segments of pyramidal cells, which lie at the border of SP and SO (Buhl et al., 1994). The increased GABA_A_R γ2 clustering in SP suggests that the perisomatic synapses, in particular, are strengthened by HACB2-AAV transduction, although the longer rise times of spontaneous mIPSCs raise the possibility of increased GABA_A_R clustering at more distal synapses, or the addition of GABA_A_Rs of alternative compositions to the perisomatic synapses. At these synapses, GABA_A_Rs predominantly have α1β3γ2 and α2β3γ2 subunit composition (Nusser et al., 1996; Nyíri et al., 2001; Kasugai et al., 2010). The increase in postsynaptic gephyrin cluster size might attract extrasynaptic α5β3γ2 GABA_A_Rs into the postsynapse. The α5β3γ2 GABA_A_Rs are mostly extrasynaptic but can also be synaptically localized (Brünig et al., 2002; Christie et al., 2002; Schweizer et al., 2003; Alldred et al., 2005; Serwanski et al., 2006). The incorporation of α5β3γ2 GABA_A_Rs into the synapse is consistent with the longer rise times and very slow deactivation of α5β3γ2 GABA_A_Rs compared with α1β3γ2 and α2β3γ2 (Mortensen et al., 2012). In hippocampal pyramidal neurons, the α5, α2, and α1 are the three most abundant α GABA_A_R subunits, in that order (Wisden et al., 1992; Fritschy and Mohler, 1995; Nusser et al., 1996; Brünig et al., 2002; Serwanski et al., 2006; Kasugai et al., 2010). Immunofluorescence revealed that the effects of HACB2-AAV on enhancing postsynaptic GABA_A_R clustering are more subdued than the effects on gephyrin clustering. We attribute it to the fact that gephyrin clustering in these cells is largely concentrated on GABAergic synapses, while γ2-containing GABA_A_Rs are both synaptic and extrasynaptic.

We did not observe any changes in density, MFI, or size of VGAT puncta, which is consistent with previous studies indicating that CB is not synaptogenic: knocking out CB in mice, while disruptive of gephyrin clusters in certain brain regions, does not affect presynaptic GABAergic innervation (Papadopoulos et al., 2007), nor does knock-down of CBSH3– or CBSH3+ isoforms (George et al., 2021) or overexpression of CB (Chiou et al., 2011; Fekete et al., 2017). Nevertheless, we observed a left-shift in interevent interval and increase in frequency of mIPSCs in CB2SH3– overexpressing cells. Since this is not accompanied by increased presynaptic GABAergic innervation, as shown by VGAT immunofluorescence, the result is consistent with an increased probability of spontaneous vesicular GABA release from presynaptic terminals onto HACB2-AAV-transduced cells. Therefore, increased postsynaptic clustering of gephyrin/GABA_A_Rs seems to have a transsynaptic effect on spontaneous GABA release. PV+ and CCK+ basket cells in the hippocampus have distinct mechanisms for modulating presynaptic release. While CCK+ basket cell terminals are covered in cannabinoid and GABA_B_Rs (Marsicano and Lutz, 1999; Sloviter et al., 1999; Liu et al., 2019), M_2_ muscarinic and μ-opioid receptors modulate activity at PV+ basket cell terminals (Hájos et al., 1998; Drake and Milner, 2006; Freund and Katona, 2007). The increase in spontaneous mIPSC frequency is contrary to the decreased frequency observed in pyramidal cells of rat cerebral cortex transfected by IUE to overexpress CB2SH3– (Fekete et al., 2017). This difference could be attributed to: (1) being neurons in different brain areas innervated by different interneurons; or (2) the IUE study involved CB2SH3– overexpression in a sparse population of pyramidal neurons in cerebral cortex since embryonic age, which has the potential for altering synaptic function in the adult.

The possibility of enhancing synaptic GABAergic neurotransmission in hippocampal circuits is of particular interest in regards to circuits in which there is an excitation/inhibition imbalance. In the current study, we used the chemoconvulsant PTZ to induce acute seizures. The mechanism of action for PTZ is not well understood, but it has been demonstrated to cause calcium and sodium influx in neurons, thereby resulting in depolarization (Papp et al., 1987). PTZ been used to model acute seizures with a single dose, and to study epileptogenesis in chemical kindling models with multiple subthreshold doses. In VGLUT1-cre mice injected with HACB2-AAV, latency to PTZ-induced seizure events was increased and mice were less likely to develop severe seizures, thereby demonstrating that enhancing the strength of GABAergic synaptic inhibition through HACB2-AAV transduction provided protection against convulsions. Our results bring attention to CB as a possible target for therapeutic intervention. They suggest that enhancing CB expression or enhancing the activity of existing endogenous CB in a selected population of hyperactive hippocampal neurons may decrease their activity and prove useful in ameliorating seizures originating in hippocampal structures such as temporal lobe epilepsy.

In VGAT-cre mice, we quantified interneurons with soma residing within the SP. While the majority of these cells are PV+ basket cells, there are many additional interneuron types whose soma can be found within or immediately adjacent to the SP, including CCK+ basket cells, PV+ bistratified or chandelier cells, ivy cells, and vasointestinal active peptide (VIP)+ type 1 or three interneuron selective interneurons (Klausberger, 2009; Pelkey et al., 2017). Many additional interneuron types were transduced in the SO and SR that may be bistratified cells, oriens–lacunosum moleculare cells, or CCK+/VIP+ basket cells (Sik et al., 1995; Baude et al., 2007). The vast majority of boutons from pyramidal layer interneurons terminate on pyramidal cells within the SP or onto the soma of other basket cells or bistratified cells within the SP (Sik et al., 1995; Földy et al., 2010). CB may be differentially expressed between various interneuron types. It has been shown that PV+ interneurons in the hippocampus and cerebral cortex contain higher levels of endogenous CB (Arhgef9) transcripts and protein than other interneurons (Paul et al., 2017; George et al., 2021). We found that transduction with the HACB2-AAV resulted in increased expression of CB in transduced interneurons compared with control EGFP-AAV transduced interneurons.

Transduction of interneurons with HACB2-AAV resulted in roughly two-fold increases in both size and density of perisomatic gephyrin and modest increases in perisomatic GABA_A_R clusters. This effect was not accompanied by any changes to size, density, or MFI of presynaptic VGAT input onto the soma, indicating that CBSH3– overexpression does not recruit additional GABAergic innervation to these cells. To maintain consistency in our analysis, we restricted ourselves to quantification in cells within the center of SP. However, nearly every HACB2-AAV-transduced CA1 interneuron, including those in SO and on the perimeter of SR, displayed a noticeably strong perisomatic ring of gephyrin clusters. This was rarely seen in non-transduced interneurons of the contralateral CA1 or in EGFP-AAV-transduced CA1 interneurons. However, strong diffuse or punctate extrasynaptic immunoreactivity with the GABA_A_R γ2 antibody was frequently seen in CA1 interneurons, in VGAT-cre as well as VGLUT1-cre mice, and in both HACB2-AAV and EGFP-AAV injected and non-injected hemispheres, indicating that the γ2-containing GABA_A_Rs in interneurons are not only synaptic but also extrasynaptic.

The results of HACB2-AAV injection in VGAT-cre mice indicates that our approach can also be used for targeting GABAergic interneurons for the conditional overexpression of CB2SH3–. However, because of the heterogeneity of the transduced interneurons and the diversity of synaptic inhibitory GABAergic inputs from other interneurons, the VGAT-cre mice are not adequate to properly interrogate the functional outcomes, which would be hard if not impossible to interpret. Instead, CB overexpression can be tailored to target-specific classes of interneurons in the hippocampus or any region of the brain by using selected interneuron-type-specific cre-driver mouse lines (i.e., PV-cre, CCK-cre, Calbindin2-cre, SOM-Cre, and VIP-cre among others). These future studies are beyond the scope of this article.

Enhancement of inhibitory synapses have also been accomplished by overexpression of NL2, a cell-adhesion molecule found selectively at inhibitory postsynapses which interacts with and activates CBSH3+ (Poulopoulos et al., 2009; Soykan et al., 2014). It has also been demonstrated to interact with gephyrin and CB forming a tri-partite complex at GABAergic synapses with GABA_A_R-γ2 and the transmembrane GABA_A_R accessory protein LHFPL4 or GARLH (Varoqueaux et al., 2004; Davenport et al., 2017; Yamasaki et al., 2017). Two studies have investigated AAV-mediated overexpression of NL2 in the hippocampus. In one study, overexpression of NL2 results in increased gephyrin clusters and VGAT+ puncta by confocal microscopy immunofluorescence and an increase in the gephyrin, VGAT and GABA_A_R-γ2 subunit protein expression by western blot, suggesting a strengthening inhibitory synapses in the transduced neurons (Van Zandt et al., 2019). In the other study, increased NL2 expression leads to increased mRNA levels for GAD65, but no increase in neurexin-1, gephyrin, or GAD67 mRNAs (Kohl et al., 2013). Interestingly, the two AAV-mediated NL2-overexpression studies also revealed alterations to behavior, notably, reductions in social aggression and dominance behaviors but also diminished preference for social novelty. In another study, overexpression of NL2 in cortical pyramidal neurons after *in utero* electroporation shows an increase in VGAT which were associated to gephyrin clusters (Fekete et al., 2015). An increase in VGAT, but no change in gephyrin was reported in transgenic animals overexpressing NL2 (Hines et al., 2008). In the same study, it was determined that pyramidal neurons in the prefrontal cortex of these transgenic mice had increased mIPSC frequency but showed no effect on the amplitude of mIPSCs (Hines et al., 2008; Fekete et al., 2015). It is worth noting that in the two AAV-NL2 studies, NL2 was simultaneously overexpressed in both glutamatergic and GABAergic neurons. The NL2 overexpression studies strongly suggest that NL2 overexpression in neurons enhances GABAergic synaptic transmission and that if targeted to specific neurons might also be effective for seizure suppression.

The HACB2-AAV and described methodology can be generally applied for selective *in vivo* enhancement of GABAergic synaptic transmission in any neuronal type and brain region, by using the expanding collection of available Cre-driver mouse lines. Moreover, our DIO-AAV could also be used for the overexpression of CBSH3– in selected types of neurons in wild-type non-transgenic animal species besides mouse, by co-injecting it with another AAV carrying Cre-recombinase under the control of a neuron-type-specific modified promoter. This has been accomplished in rat and monkey (Mikhailova et al., 2016; Stauffer et al., 2016; Zalocusky et al., 2016).

## Synthesis

Reviewing Editor: Nathalie Rouach, College of France Decisions are customarily a result of the Reviewing Editor and the peer reviewers coming together and discussing their recommendations until a consensus is reached. When revisions are invited, a fact-based synthesis statement explaining their decision and outlining what is needed to prepare a revision will be listed below. The following reviewer(s) agreed to reveal their identity: Sabine Levi. Note: If this manuscript was transferred from JNeurosci and a decision was made to accept the manuscript without peer review, a brief statement to this effect will instead be what is listed below.

Reviewer 1:

eN-TNWR-0561-20X entitled “Selective overexpression of Collybistin in mouse hippocampal pyramidal cells enhances GABAergic neurotransmission and protects against PTZ-induced seizures” examines viral-mediated overexpression of an isoform of collybistin (CB) lacking SH3 domain CBS3-, within the hippocampus. The goal of the study was to determine whether overexpression of this isoform enhanced GABAergic postsynaptic contacts, increased spontaneous mini inhibitory postsynaptic potentials in slices from mice with overexpression of the CBSH3- isoform of collybistin, and functionally increased inhibitory neurotransmission in the PTZ model of seizures.

To determine whether increased expression of the CBSH3- isoform of collybistin in glutamatergic neurons was sufficient to enhance inhibitory neurotransmission, they used VGAT-cre mice and showed that AAV-mediated collybistin overexpression resulted in increased size and number of gephyrin clusters throughout the pyramidal layer in CA1 region of the hippocampus, compared to control AAV transduced cells. The authors also found an increase in the size of g2GABAAR receptor subunit clusters, but no changes in their density. The authors also did not find a significant change in VGAT+ synaptic puncta size, density or fluorescence intensity, suggesting that overexpression of the CBSH3- isoform of collybistin did not alter the number or size of presynaptic inhibitory synapses.

Similarly, conditional AAV-mediated overexpression of the CBSH3- isoform of collybistin in inhibitory GABAergic interneurons in CA1 increased the density and size of gephyrin clusters on interneurons, but they found only modest increases in g2GABAAR receptor subunit clusters on inhibitory interneurons. They found no changes in size, density of VGAT+ terminals onto the transduced interneurons, again suggesting that all of the effects were localized to the postsynaptic side of GABAergic synaptic contacts.

Patch-clamp electrophysiological recordings demonstrated stronger inhibitory synaptic neurotransmission, as shown by increased number and amplitudes of mIPSCs in transduced pyramidal neurons, lower interevent intervals, and higher frequencies of mIPSCs. These findings, together with the immunofluorescent staining results suggesting selective changes post-synaptically, were interpreted as indicating a higher probability of presynaptic release of neurotransmitter from GABAergic terminals. Thus, the authors suggest that the changes induced to postsynaptic structures were affecting presynaptic release probability.

Additional experiments support the functional interpretation by showing in the PTZ model, VGLUT-cre mice with overexpression of the CBSH3- isoform had fewer higher intensity seizures, as scored behaviorally using the Racine scale, and increased latency for Racine scale 1 or 2 seizures.

Several issues could be addressed through textual revisions to improve the overall quality of the manuscript and reproducibility of the experiments.

1. Authors should revise the subheadings for sections describing overexpression of the CBSH3- isoform of collybistin to provide a summary of the major findings in these sections, similar to the subheading for the electrophysiology and PTZ seizure experiments. This would help the flow of the manuscript.

2. Authors provide very little context for their findings in comparison to recent work that used very similar viral approaches and immunochemical analyses to examine the effects of overexpression of other members of the postsynaptic protein scaffold. Some additional discussion of this literature would help drive a better understanding of the roles of different proteins in the scaffold in recruiting other proteins to the complex, as well as in enhancing GABAergic neurotransmission. For example, the present findings contrast with work examining overexpression of neuroligin 2 (Van Zandt et al. Behav Brain Res 2019 Apr 19;362:7-20. doi: 10.1016/j.bbr.2018.12.052). In this previous study, while viral mediated overexpression was not conditional and both glutamatergic and GABAergic neurons of the hippocampus were transduced with a vector leading to overexpression of neuroligin2, the authors report very similar findings, including increased gephyrin and g2GABAAR receptor subunit clusters in hippocampal pyramidal neurons. However, in contrast to the current work, the authors also reported increased numbers of VGAT+ puncta. For example, would overexpression of neuroligin be even more effective for seizure suppression?

3. The number of mice used for immunohistochemical, electrophysiological and PTZ studies should be described, as well as the number of animals that were excluded for different reasons. While the N for the statistical studies is included in Table 1, please clarify how many different sections/mouse were analyzed. Additionally, information about mortality during any of the procedures should be included.

Reviewer 2:

This work addresses the effect of an overexpression of a constitutively active isoform of collybistin CB2SH3- in either pyramidal cells or interneurons of the CA1 region of the hippocampus on GABAergic inhibition (organization and strength of inhibitory synapses) in order to know if this could at term constitute a strategy to elevate inhibition in diseases with altered GABAergic transmission such as epilepsy. For this purpose, they injected HA-tagged CB2 adenoviruses in dorsal hippocampi of VGLUT1-IRES-cre mice or VGAT-IRES-cre mice, in order to overexpress CB2 in pyramidal glutamatergic cells or in GABAergic interneurons of the hippocampus. They found that expressing CB2 in pyramidal cells and interneurons do increase gephyrin cluster size in both type of cells with only partial effect on the clustering of the gamma2 subunit of the GABAA receptor (layer and cell variable effects). Then they show that expressing CB2 in CA1 pyramidal cells increases GABAA receptor-mediated synaptic currents and reduces PTZ-induced seizures in infected VGLUT1-IRES-cre mice.

Although the role of CB on GABAergic synapses has been studied previously, it is not known whether there is a synapse specific effect of CB2. However, it is unfortunate that the authors did not quantify CB staining in order to correlate their findings with an effective CB overexpression. Moreover, the functional impact of CB2 overexpression on the strength of GABAergic synapses and on the incidence of seizures is only partially addressed in this manuscript. Either the authors investigate this in both interneurons and pyramidal cells or they explain why they did it solely in CB2-expressing pyramidal cells (due to a lack of CB overexpression and/or function in interneurons?).

I believe that these two main points should be addressed before publication.

To validate their approach, they performed immunofluorescence of the HA tag and CB in hippocampal slices to determine whether the viral expression of CB2SH3- is selective of the targeted cell (Pyr cell or Interneuron) and whether this strategy leads indeed to CB overexpression in the appropriate cells (Fig. 1). This is a critical point and the authors only show a single image of CB2 staining in pyramidal cells or interneurons from which they conclude that these cells do overexpress CB. Since all the work is based on this statement, they need to produce a rigorous quantitative analysis of HA and CB expression (from several ROIs and animals, as usually done) to demonstrate significant overexpression of CB compared to the endogenous protein in the different cell types. Quantification should be done in pyramidal cells as well as interneurons in both AAV-infected VGlut1 and VGAT mice to show selectivity (overexpression of CB2 in pyramidal cells but not in interneurons in VGLUT1-IRES-cre mice and vice versa in VGAT-IRES-cre mice).

In VGLUT1-IRES-cre mice, it is curious that the density and size of synaptic gephyrin clusters increase in pyramidal cell soma and dendrites (SO, SP and SR layers of the CA1 region display larger gephyrin clusters) while the size of GABAAR gamma2 clusters increases only in the soma of pyramidal cells (SP layer). Do the authors have an explanation for this discrepancy? What about other receptor subunits? GABAA receptor alpha2 and alpha1 subunits directly bind to gephyrin at the difference of the gamma2 subunit and these subunits have been associated with different interneuron-principal cell synapses (Parvalbumin and CCK interneurons making synapses onto soma or dendrites of pyramidal cells). This questions about a synapse selective impact of CB2 overexpression on GABAergic synapses.

In VGAT-IRES-cre mice, they show no significant effect on the number and size of GABAA receptor gamma2 clusters in any of the layers analyzed while they see increased gephyrin clustering. Does it mean that it is not possible to overexpress CB2 in interneurons or that there is no functional impact of CB overexpression?

Then they studied the functional impact of CB overexpression by analyzing synaptic currents and PTZ-induced seizure susceptibility/severity. This was assessed in VGLUT1-IRES-cre mice but not in VGAT-IRES-cre mice. This is unexpected because they compared both type of cells in the first part of the manuscript without concluding on a cell/synapse specific effect of CB2 overexpression. Does it mean that there was no effect on synaptic currents and ictogenesis in VGAT animals? Whatever the results, it will be good to know. What about endogenous CB2 expression in interneurons vs pyr cells in the first place? Do we know if interneurons do express endogenous CB2? If not this may help to understand the discrepancy between the two cell types.
